# Epidemiology and injectable antiseizure medication treatment patterns of seizure patients treated in United States hospitals

**DOI:** 10.3389/fneur.2022.941775

**Published:** 2022-09-12

**Authors:** Silky Beaty, Ning Rosenthal, Julie Gayle, Prashant Dongre, Kristen Ricchetti-Masterson, Denise H. Rhoney

**Affiliations:** ^1^UCB Pharma, Smyrna, GA, United States; ^2^PINC AI™ Applied Sciences, Premier Inc., Charlotte, NC, United States; ^3^Sarepta Therapeutics, Cambridge, MA, United States; ^4^UNC Eshelman School of Pharmacy, University of North Carolina, Chapel Hill, NC, United States

**Keywords:** seizures, injectable antiseizure medication, hospitals, prevalence, outcomes

## Abstract

**Objective:**

This study aimed to examine the epidemiology of seizures, clinical outcomes, and antiseizure medication treatment patterns among seizure patients treated in United States hospitals.

**Design:**

A retrospective cross-sectional study was conducted using data from a large geographically diverse hospital discharge database.

**Setting:**

860 acute care hospitals in the United States.

**Participants:**

Patients aged ≥18 years with an outpatient emergency department or inpatient visit between 1 July 2016–31 December 2019 were included.

**Intervention:**

None.

**Main outcomes and measures:**

Key outcomes included prevalence of seizure, seizure type, admission point of origin, intensive care unit admission, discharge status, and injectable antiseizure medication utilization. Seizures were identified by the International Classification of Disease, Tenth Revision, Clinical Modification diagnosis codes.

**Results:**

Among 36,598,627 unique emergency department outpatients (72,372,464 outpatient visits) and 16,543,592 unique inpatients (24,923,489 inpatient admissions) analyzed, seizure was present in 2.1% of outpatients (1.87% of outpatient visits) and 4.9% of inpatients (4.8% of inpatient admissions). In overall seizure patients, 49.1% were unclassified, 4.4% had generalized onset, 2.9% had focal onset, and 42.8% were categorized as other (including 38.5% with convulsion). Among seizure-associated inpatient admissions, <1% were transferred directly from skilled nursing facility or other long-term care facilities but 22.7% were discharged to such facilities. Nearly a third (31%) of all inpatients were admitted to ICU. About 88.3% of patients with injectable ASM use had monotherapy, 4.6% had polytherapy with 1 day or multiple non-consecutive days of overlap, and 7.0% had polytherapy with ≥2 consecutive days of overlap. The percentage of patients with no step down to any oral ASM ranged between 34.0–57.0%.

**Conclusions:**

Seizures affect a substantial number of hospital-based emergency department outpatient and inpatient encounters and are associated with poor clinical outcomes and significant healthcare burden. Concomitant use of injectable ASMs is uncommon and a high percentage of IV ASM users with a diagnosis of seizure had no step down to oral therapy.

**Relevance:**

The study findings may inform clinicians and hospital decision makers about current clinical practice and burden of seizures and identify areas to improve overall outcomes for patients with seizures.

## Introduction

Seizures are prevalent among hospital patients with rates varying by hospital setting. A study using the 1993–2003 National Hospital Ambulatory Medical Care Survey (NHAMCS) data showed that seizures accounted for about 1% (1 million visits annually) of all emergency department (ED) visits in the United States (US) (1). According to the Agency for Healthcare Research and Quality, seizures or epilepsy were identified in approximately 3.6% of the total 39.2 million hospitalizations in 2005 (2). Within the medical intensive care unit (ICU), about 10% of patients experience seizures; this rate increases to as much as 33% in the neurocritical care unit (NCCU) (3, 4). However, these estimates are dated, and there is a pressing need to assess the current prevalence of seizures, characteristics of seizure patients, their treatment patterns, and clinical outcomes within the US hospital setting.

Seizures treated in hospitals may either be due to recurrence or exacerbation of pre-existing epilepsy or be caused by diseases that directly or indirectly affect a patient's brain such as stroke, trauma, brain tumor, infection, or metabolic disturbances (5). Seizures have been associated with poor clinical outcomes among patients treated in the hospital setting (6). Timely identification and appropriate treatment of seizures are key to reducing disease-related healthcare burden and improving patient outcomes (7–9). Injectable antiseizure medications (ASMs) may provide rapid delivery and complete (intravenous) or nearly complete (intramuscular) bioavailability and are the primary method of seizure management in acute care hospitals (5, 10). However, with multiple injectable ASMs approved by the US Food and Drug Administration during the past three decades, the choice of injectable ASMs has evolved and become more complex, especially in the hospital setting (11).

Injectable ASM therapy in hospitals is often used either to rapidly control acute seizures and/or for treating patients with primary or secondary seizure diagnosis who cannot take the oral form of ASMs (10). This is important as recurrent seizures are common in hospital patients (5), which can lead to potential complications and impact patient outcomes (12–15). Injectable ASMs can be used as monotherapy or as combination therapy in hospitals (16, 17). There is a paucity of data on how injectable ASMs are being used in US hospitals. Understanding the ASM utilization pattern and patient journey within the hospital setting may inform clinicians about the current clinical practice and identify areas to improve overall outcomes for patients with seizures.

Using geographically diverse hospital discharge data from 860 hospitals, this study aimed to understand the epidemiology, clinical outcomes, and utilization patterns of injectable ASMs among patients with seizure diagnosis in US hospitals.

## Materials and methods

We conducted a retrospective observational study utilizing data from the Premier PINC AI™ Healthcare Database (PHD) to estimate the prevalence of seizures and assess patient and hospital characteristics, patient journey, clinical outcomes and utilization patterns of injectable ASM within hospital setting. The PHD is a large, geographically diverse, hospital-based, service-level, all-payer database containing discharge information from inpatient and hospital-based outpatient visits (12). It represents approximately 20–25% of all inpatient admissions in the US since 2000. The PHD contains patient and visit-level data from standard hospital discharge files including patient demographics, disease states, and a time-stamped log of billed items including procedures, medications, and diagnostic and therapeutic services. All data are statistically de-identified and compliant with the Health Insurance Portability and Accountability Act. Institutional review board approval for this study was not required, based on US Title 45 Code of Federal Regulations, Part 46, because the study used existing de-identified hospital discharge data, and recorded information could not be identified directly or through identifiers linked to individuals. No informed consent of study participants was pursued due to the nature of the deidentified data.

Adult patients having an ED outpatient visit or an inpatient admission discharged between 1 July 2016–31 December 2019 from a PHD hospital were analyzed. Seizures were identified as having a principal or secondary International Classification of Disease, Tenth Revision, Clinical Modification [ICD-10-CM] discharge diagnosis code of seizure (see Supplementary Table 1 for code list). Patients with missing age were excluded.

Patient demographics included age, sex, race, and ethnicity. Seizure-specific comorbidities were defined using the St. Germaine-Smith's assessment scale, which included congestive heart failure, peripheral vascular disease, renal disease, moderate to severe liver disease, metastatic cancer, brain tumor, solid tumor without metastasis, paraplegia and hemiplegia, aspiration pneumonia, dementia, pulmonary circulation disease, cardiac arrhythmias, hypertension, and anoxic brain injury (see Supplementary Table 2 for diagnosis codes) (18). Admission type, admission source, discharge disposition, and primary payer type were also assessed. Hospital characteristics included teaching status, urban or rural populations served, geographical region, and bed size.

Concomitant injectable ASM use was assessed among inpatient visits with seizure diagnosis and a hospital length of stay ≥ 2 days using billing data. Utilization was categorized as monotherapy (had a maximum of one injectable ASM on any given day during index hospitalization) or polytherapy. Polytherapy was defined as (1) use of two or more IV ASMs with 1 day or non-consecutive days of overlap, or (2) use of two or more IV ASMs together on two or more consecutive days. Polytherapy was further categorized as 2, 3, and 4+ injectable ASMs used concomitantly, which referred to the maximum number of injectable ASMs in the combination used on consecutive days.

Injectable ASM treatment formulation changes were assessed among inpatient visits with seizure diagnosis, length of stay ≥2 days and having monotherapy of injectable ASM treatment. Commonly used IV ASMs assessed included levetiracetam, fosphenytoin, phenytoin, sodium valproate, lacosamide, brivaracetam, and phenobarbitone. Step down from IV form to its own oral form was assessed for each of the selected IV ASMs except valproate and fosphenytoin. For brivaracetam and levetiracetam, step down from IV to other oral drugs was also assessed. Step down means that there is an oral form of ASM given on or after the last day of an IV ASM formulation. When there were multiple IV ASMs used during the visit, we only assessed the step-down pattern for the last IV ASM used.

Prevalence of use for three injectable benzodiazepine (BZD) drugs was assessed and reported by type of visit. The medications assessed included lorazepam IV, diazepam IV, and midazolam IM and IV (see Supplementary Table 3 for list of IV ASMs).

### Statistical analysis

We first estimated the prevalence of patients and visits with diagnoses of seizures for ED outpatient and inpatient visits, respectively. We then assessed the patient and hospital characteristics. Lastly, we examined the patient journey, clinical outcomes and injectable ASM utilization patterns. Data measured on a continuous scale were expressed as mean, standard deviation, median, and interquartile range (IQR). Categorical data were expressed as counts and percentages of patients in each category. Patient characteristics were reported by type of visit (ED outpatient vs. inpatient). Chi-square test were used to test for statistical differences between groups for categorical variables. Two sample comparisons were evaluated using a *t*-test (for normally distributed data) or Wilcoxon Rank Sum test (when data was not normally distributed) for continuous variables. All statistical analyses were performed using SAS v9.4 (Cary, NC), and a two-sided *p* < 0.05 was considered statistically significant.

## Results

### Prevalence of patients or visits with diagnoses of seizure and type of seizure

A total of 36,598,627 unique ED outpatients who had 72,372,464 discrete ED outpatient visits and 16,543,592 unique inpatients who had 24,923,489 discrete inpatient admissions were analyzed. An estimated 2.1% of unique ED outpatients (1.9% of ED outpatient visits) and 4.9% of inpatients (4.8% of inpatient visits) had a discharge diagnosis of seizure. The observed yearly prevalence in both ED outpatients and inpatients was similar across the study period.

As shown in eFigure, 52% of inpatient visits had seizure type as unclassified, and more than a third (37%) fell in the “other” category. Focal seizures and generalized seizures accounted for 5% of each. While among ED outpatient visits with a seizure diagnosis as shown in eFigure, 46% had seizure type unclassified, and 49% classified as other seizure type; Focal seizures accounted for 1%; and generalized accounted for 3% (Supplementary Figure S1).

### Characteristics of seizure patients and discharge disposition

Among the 1,359,891 unique patients with a principal/secondary discharge diagnosis of seizure, 52% were inpatients and 48% were ED outpatients. Compared to ED outpatients, inpatients were older (57.9 ± 18.6 vs. 44.9 ± 18.1 years), less likely to be female (50.6% vs. 52.4%) and Hispanic (6.4% vs. 7.1%), and more likely to be White (70.3% vs. 68.8%) and have Medicare as primary payer (53.8% vs. 30.0%) (all *p* < 0.001) (Table 1).

**Table 1:** Patient and hospital characteristics among patients with a seizure diagnosis during index hospital visit.

	**Total**		**ED outpatients**		**Inpatients**		**p**
	***n***	**%**	***n***	**%**	***n***	**%**	
**# of unique patients**	**1,359,891**	**100.0%**	**652,769**	**48.0%**	**707,122**	**52.0%**	
**Age (years, Mean** **±Std. Dev.)**	51.7	±19.5	44.9	±18.1	57.9	±18.6	<0.0001
**Age group (years)**							
18–34	327,523	24.1%	228,286	35.0%	99,237	14.0%	<0.0001
35–49	294,440	21.7%	173,192	26.5%	121,248	17.1%	
50–64	356,333	26.2%	148,604	22.8%	207,729	29.4%	
65–74	190,976	14.0%	56,131	8.6%	134,845	19.1%	
75–84	125,637	9.2%	31,321	4.8%	94,316	13.3%	
85+	64,982	4.8%	15,235	2.3%	49,747	7.0%	
**Sex**							
Male	659,932	48.5%	310,483	47.6%	349,449	49.4%	<0.0001
Female	699,583	51.4%	342,080	52.4%	357,503	50.6%	
Unknown	376	0.0%	206	0.0%	170	0.0%	
**Race**							
White	946,016	69.6%	448,992	68.8%	497,024	70.3%	<0.0001
Black	251,584	18.5%	128,589	19.7%	122,995	17.4%	
Other	162,291	11.9%	75,188	11.5%	87,103	12.3%	
**Ethnicity**							
Hispanic	91,667	6.7%	46,304	7.1%	45,363	6.4%	<0.0001
Non–Hispanic	982,585	72.3%	472,221	72.3%	510,364	72.2%	
Unknown	285,639	21.0%	134,244	20.6%	151,395	21.4%	
**Primary payer type**							
Commercial	271,988	20.0%	149,828	23.0%	122,160	17.3%	<0.0001
Medicare	576,817	42.4%	196,059	30.0%	380,758	53.8%	
Medicaid	338,936	24.9%	193,749	29.7%	145,187	20.5%	
Other payer	172,150	12.7%	113,133	17.3%	59,017	8.3%	
**Hospital setting**							
Urban	1,175,141	86.4%	550,027	84.3%	625,114	88.4%	<0.0001
Rural	184,750	13.6%	102,742	15.7%	82,008	11.6%	
**Teaching status**							
Teaching	632,564	46.5%	264,177	40.5%	368,387	52.1%	<0.0001
Non–teaching	727,327	53.5%	388,592	59.5%	338,735	47.9%	
**Census regions**							
Midwest	304,757	22.4%	152,158	23.3%	152,599	21.6%	<0.0001
Northeast	226,622	16.7%	92,224	14.1%	134,398	19.0%	
South	637,257	46.9%	304,763	46.7%	332,494	47.0%	
West	191,255	14.1%	103,624	15.9%	87,631	12.4%	
**Bed size**							
<100	106,377	7.8%	75,585	11.6%	30,792	4.4%	<0.0001
100–199	200,354	14.7%	114,490	17.5%	85,864	12.1%	
200–299	219,242	16.1%	115,045	17.6%	104,197	14.7%	
300–499	383,279	28.2%	174,955	26.8%	208,324	29.5%	
500+	450,639	33.1%	172,694	26.5%	277,945	39.3%	
**Admission type**							
Emergency	1,022,052	75.2%	524,987	80.4%	497,065	70.3%	<0.0001
Urgent	134,477	9.9%	25,949	4.0%	108,528	15.3%	
Elective	91,511	6.7%	5,836	0.9%	85,675	12.1%	
Trauma center	13,305	1.0%	3,193	0.5%	10,112	1.4%	
Other	98,546	7.2%	92,804	14.2%	5,742	0.8%	
**Discharge status**							<0.0001
Expired	41,893	3.1%	1,359	0.2%	40,534	5.7%	
Home	911,898	67.1%	569,463	87.2%	342,435	48.4%	
Hospice	25,135	1.8%	901	0.1%	24,234	3.4%	
SNF, ICF, rehabilitation or LTCF	173,983	12.8%	13,738	2.1%	160,245	22.7%	
Transferred to another acute care facility	164,061	12.1%	43,973	6.7%	120,088	17.0%	
Other	42,921	3.2%	23,335	3.6%	19,586	2.8%	
**Type of seizure diagnosis**							<0.0001
Principal diagnosis	391,963	28.8%	271,181	41.5%	120,782	17.1%	
Secondary diagnosis	967,928	71.2%	381,588	58.5%	586,340	82.9%	
**Comorbidities (St. Germaine Smith's Assessment Scale)**							
Congestive heart failure	123,480	9.1%	17,664	2.7%	105,816	15.0%	<0.0001
Peripheral vascular disease	65,710	4.8%	12,875	2.0%	52,835	7.5%	<0.0001
Renal disease	43,432	3.2%	7,822	1.2%	35,610	5.0%	<0.0001
Moderate or severe liver disease	13,953	1.0%	1,390	0.2%	12,563	1.8%	<0.0001
Metastatic cancer	31,528	2.3%	4,332	0.7%	27,196	3.8%	<0.0001
Brain tumor	22,349	1.6%	4,387	0.7%	17,962	2.5%	<0.0001
Solid tumor without metastasis	47,388	3.5%	8,225	1.3%	39,163	5.5%	<0.0001
Paraplegia and hemiplegia	6,258	0.5%	1,221	0.2%	5,037	0.7%	<0.0001
Aspiration pneumonia	60,225	4.4%	2,322	0.4%	57,903	8.2%	<0.0001
Dementia	112,965	8.3%	23,224	3.6%	89,741	12.7%	<0.0001
Pulmonary circulation disorders	42,887	3.2%	4,860	0.7%	38,027	5.4%	<0.0001
Cardiac arrhythmias	138,548	10.2%	39,289	6.0%	99,259	14.0%	<0.0001
Hypertension	652,081	48.0%	207,149	31.7%	444,932	62.9%	<0.0001
Anoxic brain injury	25,378	1.9%	642	0.1%	24,736	3.5%	<0.0001
**Mean comorbidity index** (Mean ± Std. dev.)	1.3	±1.8	0.6	±1.1	1.9	±2.0	<0.0001

SNF, skilled nursing facility; ICF, intermediate care facilities; LTCF, long-term care facilities; ED, emergency department; Std. Dev, standard deviation.

Hospital and other patient characteristics were also significantly different between ED outpatients and inpatients. A higher percentage of inpatients than ED outpatients were treated in urban (88.4% vs. 84.3%), teaching (52.1% vs. 40.5%), and large hospitals with 500+ beds (39.3% vs. 26.5%) (all *p* < 0.001). A significantly higher percentage of inpatients with seizure diagnosis died (5.7% vs. 0.2%), were discharged to hospice (3.4% vs. 0.1%) or to skilled nursing facilities (SNF), intermediate care facility (ICF), rehabilitation, or long-term care facility (LTCF) (22.7% vs. 2.1%) or were transferred to another acute care facility (17.0% vs. 6.7%) than ED outpatients (all *p* < 0.001) (Table 1).

Among seizure-associated visits, seizure was principal diagnosis for 42% of ED outpatient visits compared to 17% of inpatient admissions (*p* < 0.001). The most common comorbidities for the overall seizure patients were hypertension (48.0%), congestive heart failure (9.1%), cardiac arrhythmia (10.2%), and dementia (8.3%). All rates were higher in inpatients than in ED outpatients (Table 1). The mean comorbidity index score was 1.9 ± 2.0 for inpatients compared to 0.6 ± 1.1 for ED outpatients (*p* < 0.001).

### Patient journey in hospital

**Figure 1:**
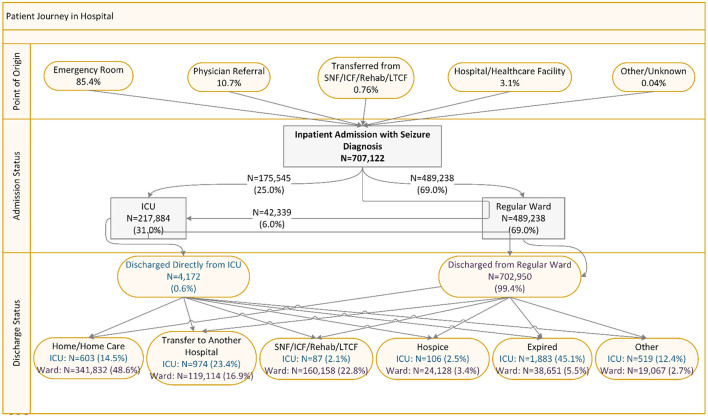
Patient journey among inpatient admissions with seizure diagnosis. ICU, intensive care unit; SNF, skilled nursing facility; ICF, intermediate care facilities; Rehab, Rehabilitation; LTCF, long-term care facilities.

As shown in Figure 1, among the 707,122 inpatient visits with seizure diagnosis, 85.4% were admitted through ED, 10.7% were referred by physicians, 3.1% were transferred from another hospital or acute healthcare facility, and <1% were transferred from SNF/ICF/rehabilitation/LTCF. A total of 31% of inpatients were admitted to ICU (25% directly to ICU and 6% to regular ward then upgraded to ICU). Among patients observed in ICU, 0.6% were discharged directly from ICU; of which, 45.1% died, and 23.4% were transferred to another hospital. Among patients discharged from regular ward, 48.6% went home/home care, 16.9% transferred to another hospital, 22.8% went to SNF/ICF/rehabilitation/LTCF, and 5.5% died (Figure 1).

### Utilization patterns of injectable ASMs

Overall, of the 301,021 inpatients with at least one IV ASM use and with hospital LOS ≥2 days, 88.3% had monotherapy, 4.6% had polytherapy with 1 day or multiple non-consecutive days of overlap, and 7.0% had polytherapy with two or more consecutive days of overlap. Among patients with polytherapy, the majority had two IV ASMs. The concomitant IV ASM treatment patterns were consistent across primary payer types (Table 2).

**Table 2:** Concomitant injectable antiseizure medication use among inpatient visits with seizure diagnosis and two or more days of hospital stay by type of primary payer.

	**Overall**		**Commercial**		**Medicaid**		**Medicare**		**Other payer**	
	**(*****N*** = **301,021)**		**(*****N*** = **59,750)**		**(*****N*** = **63, 593)**		**(*****N*** = **141,048)**		**(*****N*** = **36,630)**	
	**N**	**%**	**N**	**%**	**N**	**%**	**N**	**%**	**N**	**%**
	**patients**		**patients**		**patients**		**patients**		**patients**	
**Monotherapy (only had 1 IV ASM on each day)**	265,844	88.3%	53,576	89.7%	56,715	89.2%	121,915	86.4%	33,638	91.8%
**Polytherapy with 1 day/multiple non-consecutive days of overlap**	13,986	4.6%	2,608	4.4%	3,026	4.8%	6,811	4.8%	1,541	4.2%
2 ASM	13,517	4.5%	2,522	4.2%	2,924	4.6%	6,579	4.7%	1,492	4.1%
3 ASM	449	0.1%	81	0.1%	97	0.2%	225	0.2%	46	0.1%
4+ ASM	20	0.0%	5	0.0%	5	0.0%	7	0.0%	3	0.0%
**Polytherapy with 2+** **consecutive days of overlap (number of days with multiple ASMs)**	21,191	7.0%	3,566	6.0%	3,852	6.1%	12,322	8.7%	1,451	4.0%
2 ASM	17,565	5.8%	2,966	5.0%	3,226	5.1%	10,171	7.2%	1,202	3.3%
3 ASM	3,208	1.1%	506	0.8%	561	0.9%	1,934	1.4%	207	0.6%
4+ ASM	418	0.1%	94	0.2%	65	0.1%	217	0.2%	42	0.1%

IV, injectable; ASM, antiseizure medication.

**Figure 2:**
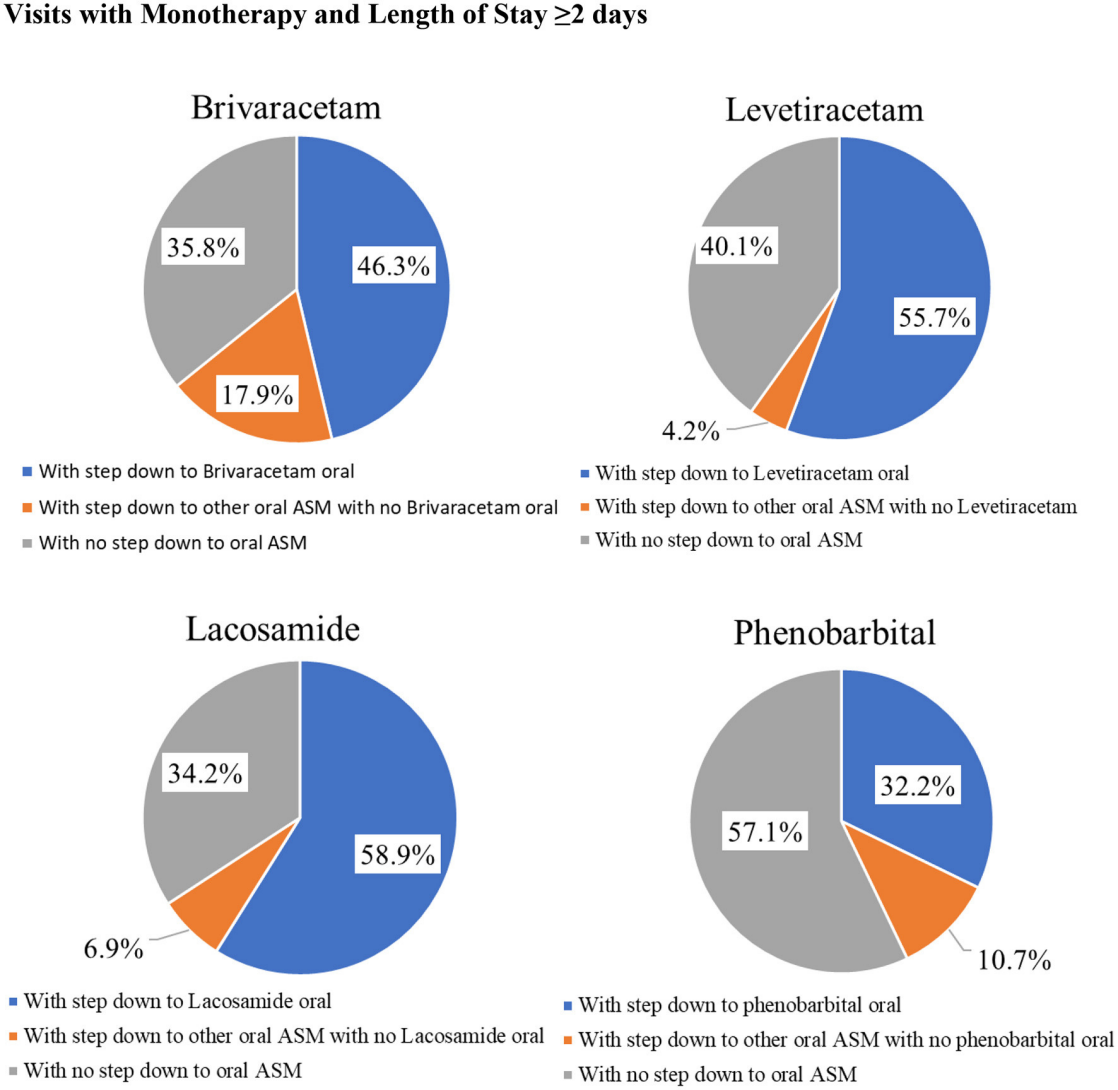
Injectable Antiseizure Medication Formulation Change Patterns among Inpatient Visits with Monotherapy and Length of Stay ≥2 days.

Among inpatients with IV ASM monotherapy and a hospital LOS ≥2 days, a higher percentage of patients stepped down to the same oral ASM than those stepping down to another oral ASM, and it was also relatively common for patients to discontinue IV ASMs with no subsequent oral ASM. Percentage of patients stepping down to the same oral ASM ranged from 32.2% for IV phenobarbitone to 59.0% for IV lacosamide; those stepping down to another oral ASM ranged from 4.0% for IV levetiracetam to 18.0% for IV brivaracetam; percentage of patients with no stepdown to any oral ASM ranged from 34.0% for IV lacosamide to 57.0% for IV phenobarbital (Figure 2).

An injectable BZD was used among 33.3% of inpatients and 9.8% of ED outpatients with a seizure diagnosis. Among patients with IV ASMs, the respective prevalence of injectable BZD use was 47.6% for inpatients and 22.1% for ED outpatients. Lorazepam was the most used injectable BZD (22.2% in inpatients and 8.4% in ED outpatients) followed by IV/IM midazolam (13.9% in inpatients and 1.1% in ED outpatients). Diazepam was only used by a small percentage of patients (Table 3). Combination use of BZD and IV ASMs was uncommon (Table 3).

**Table 3:** Frequency distribution of seizure treatment regimens of interest by type of visit with seizure diagnosis.

	**Overall**		**ED outpatient**		**Inpatient**	
**# of unique patients**	**1,359,891**	**%**	**652,769**	**%**	**707,122**	**%**
***Benzodiazepine only***						
Lorazepam IV	108,497	7.98%	38,705	5.93%	69,792	9.87%
Diazepam IV	6,213	0.46%	2,100	0.32%	4,113	0.58%
Midazolam IV/IM	61,565	4.53%	5,415	0.83%	56,150	7.94%
**Any IV/IM benzodiazepine** **+** **any one of the following IV ASM**						
Benzo+fosphenytoin/Cerebyx	6,112	0.45%	1,920	0.29%	4,192	0.59%
Benzo+levetiracetam (Keppra)	86,627	6.37%	13,899	2.13%	72,728	10.29%
Benzo+lacosamide (Vimpat)	2,065	0.15%	242	0.04%	1,823	0.26%
Benzo+depacon (valproate sodium)	2,667	0.20%	401	0.06%	2,266	0.32%
Benzo+phenobarbital/solfoton (Luminal)	1,909	0.14%	190	0.03%	1,719	0.24%
Benzo+brivaracetam (Briviact)	35	0.00%	5	0.00%	30	0.00%
***Any benzodiazepine** **+** **multiple IV ASM***						
Including briviact but with levetiracetam	190	0.01%	12	0.00%	178	0.03%
Including briviact with no levetiracetam	70	0.01%	1	0.00%	69	0.01%
Without briviact but with levetiracetam	22,463	1.65%	795	0.12%	21,668	3.06%
Without briviact or levetiracetam	1,117	0.08%	43	0.01%	1,074	0.15%
***IV ASM only (Any IV ASM use)***						
Brivaracetam (Briviact)	147	0.01%	12	0.00%	135	0.02%
Lacosamide (Vimpat)	10,304	0.76%	904	0.14%	9,400	1.33%
Levetiracetam (Keppra)	156,861	11.53%	52,859	8.10%	104,002	14.71%
Fosphenytoin (Cerebyx)	19,822	1.46%	7,034	1.08%	12,788	1.81%
Phenobarbital/solfoton (Luminal)	3,586	0.26%	562	0.09%	3,024	0.43%
Depacon (valproate sodium)	5,891	0.43%	1,306	0.20%	4,585	0.65%

ED, emergency department.

## Discussion

This large real-world study systematically examined the epidemiology of seizures and ASM utilization patterns in a nationally representative sample of hospital ED outpatient and inpatient admissions in the US during 2016–2019. Findings of this study showed that seizures affected 2.1% of ED outpatients (1.9% ED outpatient visits) and 4.9% of inpatients (4.8% of inpatient visits) treated in 860 PHD hospitals across 45 states. The prevalence of seizures found in this study was much higher than previous reports. Using the NHAMCS data during 1993–2003, Pallin et al. showed that seizures only accounted for 1% of all ED visits in the US (1). The current estimate is nearly two times of Pallin's estimate. For inpatient visits, the current estimate of seizure prevalence is 33% higher than the 2005 estimate of 3.6% from the Agency for Healthcare Research and Quality (AHRQ) study (2). Considering that the characteristics of patients/visits included in our study are comparable to those included in Pallin's study and the AHRQ study, the higher seizure prevalence in both ED outpatients and inpatients may imply increasing seizure burden in US hospitals and in the total US population in recent years. According to the 2015 National Health Interview Survey results, 1.2% of the US population reported active epilepsy with the highest number of active epilepsy cases reported than ever before (19). However, the causes of such increase remain unknown. Because the causes of seizure are diverse, it is hard to pinpoint what is causing the changes in seizure prevalence in the hospital setting. A study analyzing the increase in seizure-associated hospitalizations in 2006 deemed coding change as the primary cause of the increase (20). The switch from ICD-9 codes to ICD-10 codes on 1 October 2015 in the US might have played a role in the observed changes. Further studies are needed to determine whether the increase in prevalence of seizures is due to increased detection/recording of the condition or due to true disease burden increase.

Along the patient journey within the hospital, two important findings are noteworthy. First, although <1% of patients were transferred from SNF/ICF/rehabilitation/LTCF, 22.8% of patients were discharged from regular ward to such facilities. These findings imply that patients who had hospital inpatient or outpatient visits with a primary or secondary diagnosis of seizure often had adverse clinical outcomes that require substantial healthcare resource utilization even beyond the hospital setting, which are consistent with what was reported in prior literature (21–23). Second, the prevalence of ICU admission (31%) and in-hospital mortality (5.7%) rates are high, which implies substantial burden on ICU services among patients with seizure diagnosis. In a retrospective study of patients with non-traumatic subdural hematoma, Joseph et al. found that patients with seizures had longer hospital (17.6 vs. 6.3 days) and ICU stays (9.4 vs. 3.4 days) and higher in-hospital mortality (16.1% vs. 9.2%) (24). In a cohort study of seizure-associated clinical outcomes among acute stroke patients in Canada, Burneo et al. showed that stroke patients with seizures had substantially higher 30-day and 1-year mortality rates and longer hospital length of stay than peers without seizures, which indirectly corroborated our findings (6).

We also found that among IV ASM monotherapy patients, from a third to more than half of patients with different IV ASMs did not step down to any oral form of ASM before discharge. Although (1) temporary seizures for example due to electrolyte abnormalities might not need ongoing ASM therapy and (2) some patients might fill oral ASM prescriptions at an outpatient pharmacy that are not captured in this database, for patients who do need appropriate oral ASM treatment established before discharge, the high no-step down rate could be problematic and negatively affect their outcomes.

### Limitations

This study has several limitations. First, the medication assessment was based on chargemaster descriptions, and the timing of medication billing may be different from medication administration in some records. Therefore, misclassification of concomitant use may exist. Second, misclassification of administration route may also exist due to inaccurate recording in certain cases, which may result in inaccurate estimates of formulation change. However, we expect such misclassification to be rare. Third, the identification of seizure patients was solely based on the ICD-10-CM diagnosis codes. Coding errors may exist and affect the accuracy of seizure prevalence estimates. Last, due to limitations of the data and scope of this study, we were not able to differentiate the true independent association between seizure type (i.e., acute exacerbation of epileptic seizures or seizures secondarily complicating other conditions requiring hospitalizations) and impact on overall patient outcomes.

This study also has multiple strengths. First, the study estimates are based on a large seizure patient sample from 860 hospitals from 45 states and the District of Columbia and may be generalized to the overall seizure patient population in US hospital setting. Second, the detailed time-stamped service billing info allowed us to accurately assess the patient journey within hospital setting. Third, we reported detail ASM utilization patterns within hospital and identified gaps in care, which may inform clinicians and healthcare providers to take actions to improve patient care.

## Conclusions

This large study among a nationally representative sample of seizure patients treated in US hospitals showed that over 2% of ED outpatients and nearly 5% of inpatients had seizure diagnosis, which are higher than previous reports. In addition, nearly a third of patients were admitted to ICU, and 5.7% died during inpatient hospital stay. These findings imply that hospital inpatient and outpatient admissions with a primary or secondary discharge diagnosis of seizure pose substantial and increasing burden to US hospital systems including ICU services. The high percentage of inpatients (22.7%) discharged to SNF/ICF/rehabilitation/LTCF facilities pose a great burden to non-hospital healthcare facilities and to the healthcare system overall. Concomitant use of injectable ASMs is uncommon among seizure patients treated in hospitals. A high percentage of patients with IV ASM use had no step down to oral therapy during their hospital stay, which may affect the continuum of care for patients with seizure. More research is warranted to understand the underlying causes of seizures in hospitals, whether there is a real increase in seizure-associated hospital visits and the extent of having no step down from IV ASMs among patients who really need it so that seizures can be better prevented and managed in hospitals.

## Data availability statement

The datasets presented in this article are not readily available because the database is proprietary and requires a user license. Requests to access the datasets should be directed to NR, ning_rosenthal@premierinc.com.

## Author contributions

NR and JG had full access to all the data in the study and take responsibility for the integrity of the data and the accuracy of the data analysis. SB and NR conceptualized the study objectives and study design. NR wrote the study protocol and drafted the manuscript. JG conducted the data analysis based on the study protocol. All co-authors gave input to the study design and analysis, reviewed and revised the manuscript. All authors contributed to the article and approved the submitted version.

## Funding

This study was sponsored by UCB Pharma. The funder had no role in the study conceptualization, implementation and finding interpretation.

## Conflict of interest

SB and PD are employees of UCB Pharma. KR-M was an employee of UCB Pharma at the time of the analysis and is now an employee of Sarepta Therapeutics. NR and JG are employees of Premier Inc. DR is a speaker and on the advisory board of UCB.

## Publisher's note

All claims expressed in this article are solely those of the authors and do not necessarily represent those of their affiliated organizations, or those of the publisher, the editors and the reviewers. Any product that may be evaluated in this article, or claim that may be made by its manufacturer, is not guaranteed or endorsed by the publisher.

## Supplementary material

Supplementary Figure S1 Type of seizures by type of hospital visit. ED, Emergency Department.

## References

[1] Pallin DJ Goldstein JN Moussally JS Pelletier AJ Green AR Camargo CA. Seizure visits in US emergency departments: epidemiology and potential disparities in care. Int J Emerg Med. (2008) 1:97–105. 10.1007/s12245-008-0024-4 19384659 PMC2657249

[2] Holmquist L Russo CA Elixhauser A. Hospitalizations for epilepsy and convulsions, 2005: Statistical brief #46. In: H*ealthcare Cost and Utilization Project (HCUP) Statistical Briefs*. Rockville, MD: Agency for Healthcare Research and Quality (2006). 21755631

[3] Fogang Y Legros B Depondt C Mavroudakis N Gaspard N. Yield of repeated intermittent EEG for seizure detection in critically ill adults. Neurophysiol Clin. (2017) 47:5–12. 10.1016/j.neucli.2016.09.001 27771198

[4] Schmitt SE. Utility of clinical features for the diagnosis of seizures in the intensive care unit. J Clin Neurophysiol. (2017) 34:158–61. 10.1097/WNP.0000000000000335 27571047

[5] Fields MC Labovitz DL French JA. Hospital-onset seizures: an inpatient study. JAMA Neurol. (2013) 70:360–4. 10.1001/2013.jamaneurol.337 23319087

[6] Burneo JG Fang J Saposnik G. Impact of seizures on morbidity and mortality after stroke: a Canadian multi-centre cohort study. Eur J Neurol. (2010) 17:52–8. 10.1111/j.1468-1331.2009.02739.x 19686350

[7] Abdelmalik PA Draghic N Ling GSF. Management of moderate and severe traumatic brain injury. Transfusion. (2019) 59:1529–38. 10.1111/trf.15171 30980755

[8] Betjemann JP Lowenstein DH. Status epilepticus in adults. Lancet Neurol. (2015) 14:615–24. 10.1016/S1474-4422(15)00042-3 25908090

[9] Kazl C LaJoie J. Emergency seizure management. Curr Probl Pediatr Adolesc Health Care. (2020) 50:100892. 10.1016/j.cppeds.2020.100892 33183979

[10] Patel SI Birnbaum AK Cloyd JC Leppik IE. Intravenous and intramuscular formulations of antiseizure drugs in the treatment of epilepsy. CNS Drugs. (2015) 29:1009–22. 10.1007/s40263-015-0289-0 26603741

[11] Privitera M. Current challenges in the management of epilepsy. Am J Manag Care. (2011) 17:S195–203. 21761951

[12] Ch'ang J Claassen J. Seizures in the critically ill. Handb Clin Neurol. (2017) 141:507–29. 10.1016/B978-0-444-63599-0.00028-4 28190433

[13] Strein M Holton-Burke JP Smith LR Brophy GM. Prevention, treatment, and monitoring of seizures in the intensive care unit. J Clin Med. (2019) 8:1177. 10.3390/jcm8081177 31394791 PMC6722541

[14] Vespa P Tubi M Claassen J Buitrago-Blanco M McArthur D Velazquez AG. Metabolic crisis occurs with seizures and periodic discharges after brain trauma. Ann Neurol. (2016) 79:579–90. 10.1002/ana.24606 26814699

[15] Vespa PM Miller C McArthur D Eliseo M Etchepare M Hirt D. Non-convulsive electrographic seizures after traumatic brain injury result in a delayed, prolonged increase in intracranial pressure and metabolic crisis. Crit Care Med. (2007) 35:2830–6. 10.1097/01.CCM.0000295667.66853.BC 18074483 PMC4347945

[16] Chamberlain JM Kapur J Shinnar S Elm J Holsti M Babcock L. Efficacy of levetiracetam, fosphenytoin, and valproate for established status epilepticus by age group (ESETT): a double-blind, responsive-adaptive, randomised controlled trial. Lancet. (2020) 395:1217–24. 10.1016/S0140-6736(20)30611-5 32203691 PMC7241415

[17] Sake JK Hebert D Isojärvi J Doty P De Backer M Davies K. A pooled analysis of lacosamide clinical trial data grouped by mechanism of action of concomitant antiepileptic drugs. CNS Drugs. (2010) 24:1055–68. 10.2165/11587550-000000000-00000 21090839

[18] St Germaine-Smith C Liu M Quan H Wiebe S Jette N. Development of an epilepsy-specific risk adjustment comorbidity index. Epilepsia. (2011) 52:2161–7. 10.1111/j.1528-1167.2011.03292.x 22004000

[19] Prevention CfDCa,. More Americans Have Epilepsy Than Ever Before. (2017). Avaiable online at: https://www.cdc.gov/media/releases/2017/p0810-epilepsy-prevalence.html (accessed July 1, 2021).

[20] Cárdenas VM Román GC Pérez A Hauser WA. Why U.S. epilepsy hospital stays rose in 2006. Epilepsia. (2014) 55:1347–54. 10.1111/epi.12719 25040913

[21] Beghi E. The epidemiology of epilepsy. Neuroepidemiology. (2020) 54:185–91. 10.1159/000503831 31852003

[22] Hussain SA Ortendahl JD Bentley TGK Harmon AL Gupta S Begley CE. The economic burden of caregiving in epilepsy: an estimate based on a survey of US caregivers. Epilepsia. (2020) 61:319–29. 10.1111/epi.16429 31953846

[23] Shellhaas RA. Seizure classification, etiology, and management. Handb Clin Neurol. (2019) 162:347–61. 10.1016/B978-0-444-64029-1.00017-5 31324320

[24] Joseph JR Smith BW Williamson CA Park P. Seizure correlates with prolonged hospital stay, increased costs, and increased mortality in nontraumatic subdural hematoma. World Neurosurg. (2016) 92:366–70. 10.1016/j.wneu.2016.05.033 27237418

