# Supplementary figures and images for: Epidemiology and injectable antiseizure medication treatment patterns of seizure patients treated in United States hospitals
Source: Front Neurol. 2022 Sep 12;13:941775. doi: 10.3389/fneur.2022.941775 (PMC9510892; doi:10.3389/fneur.2022.941775)

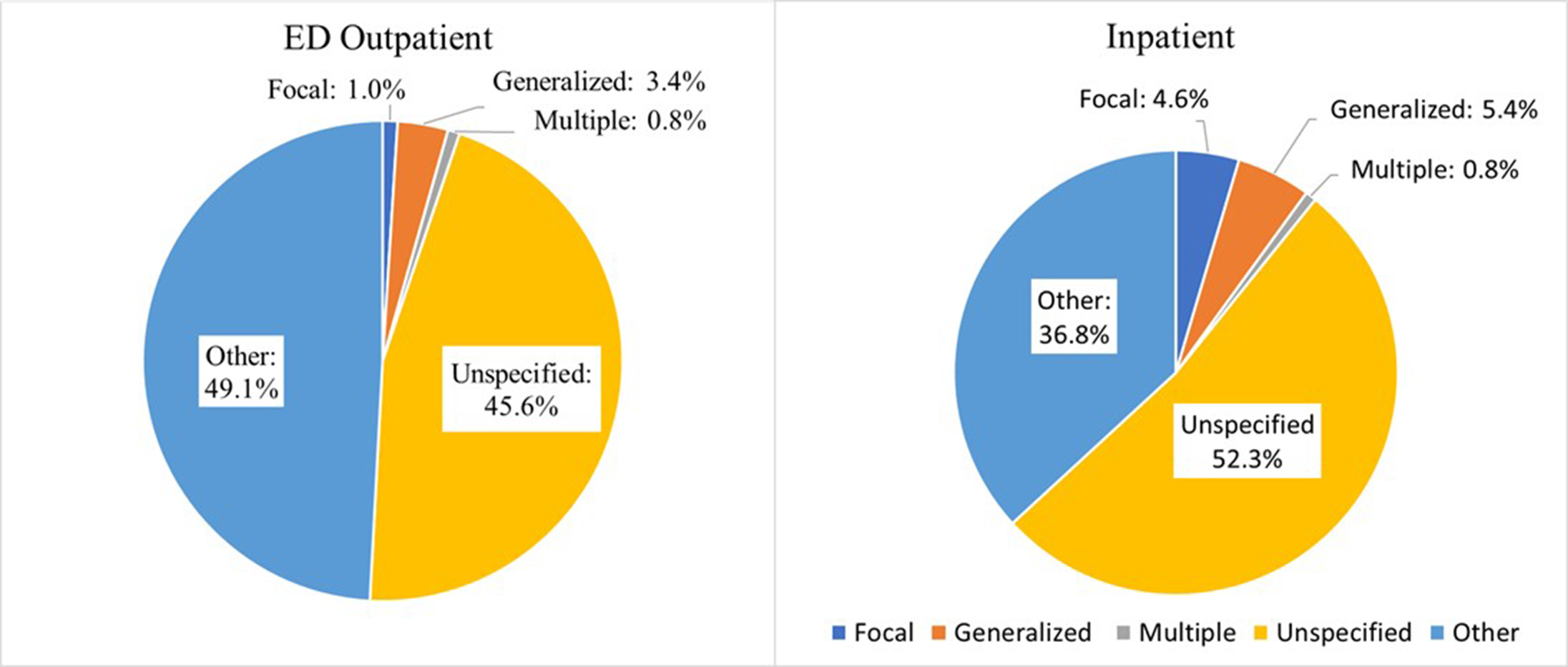

Supplement: Supplementary Figure S1 — Type of seizures by type of hospital visit. ED, Emergency Department. [file Image_1.JPEG]
