# Supplementary material for: Epidemiology and injectable antiseizure medication treatment patterns of seizure patients treated in United States hospitals
Source: Front Neurol. 2022 Sep 12;13:941775. doi: 10.3389/fneur.2022.941775 (PMC9510892; doi:10.3389/fneur.2022.941775)
Supplement: Supplementary file 2 [file Table_1.DOCX]

eTable 1: ICD-10 Diagnosis Codes for Seizures and Seizure Type

| **ICD-10 Code** | **ICD Description** | **Seizure Type/Flag** |
| --- | --- | --- |
| G40 | Epilepsy and recurrent seizures [G40] | Focal seizure |
| G40.0 | Localization-related (focal) (partial) idiopathic epilepsy and epileptic syndromes with seizures of localized onset [G40.0] | Focal seizure |
| G40.00 | Localization-related (focal) (partial) idiopathic epilepsy and epileptic syndromes with seizures of localized onset, not intractable [G40.00] | Focal seizure |
| G40.001 | Localization-related (focal) (partial) idiopathic epilepsy and epileptic syndromes with seizures of localized onset, not intractable, with status epilepticus [G40.001] | Focal seizure_SE |
| G40.009 | Localization-related (focal) (partial) idiopathic epilepsy and epileptic syndromes with seizures of localized onset, not intractable, without status epilepticus [G40.009] | Focal seizure |
| G40.01 | Localization-related (focal) (partial) idiopathic epilepsy and epileptic syndromes with seizures of localized onset, intractable [G40.01] | Focal seizure |
| G40.011 | Localization-related (focal) (partial) idiopathic epilepsy and epileptic syndromes with seizures of localized onset, intractable, with status epilepticus [G40.011] | Focal seizure_SE |
| G40.019 | Localization-related (focal) (partial) idiopathic epilepsy and epileptic syndromes with seizures of localized onset, intractable, without status epilepticus [G40.019] | Focal seizure |
| G40.1 | Localization-related (focal) (partial) symptomatic epilepsy and epileptic syndromes with simple partial seizures [G40.1] | Focal seizure |
| G40.10 | Localization-related (focal) (partial) symptomatic epilepsy and epileptic syndromes with simple partial seizures, not intractable [G40.10] | Focal seizure |
| G40.101 | Localization-related (focal) (partial) symptomatic epilepsy and epileptic syndromes with simple partial seizures, not intractable, with status epilepticus [G40.101] | Focal seizure_SE |
| G40.109 | Localization-related (focal) (partial) symptomatic epilepsy and epileptic syndromes with simple partial seizures, not intractable, without status epilepticus [G40.109] | Focal seizure |
| G40.11 | Localization-related (focal) (partial) symptomatic epilepsy and epileptic syndromes with simple partial seizures, intractable [G40.11] | Focal seizure |
| G40.111 | Localization-related (focal) (partial) symptomatic epilepsy and epileptic syndromes with simple partial seizures, intractable, with status epilepticus [G40.111] | Focal seizure_SE |
| G40.119 | Localization-related (focal) (partial) symptomatic epilepsy and epileptic syndromes with simple partial seizures, intractable, without status epilepticus [G40.119] | Focal seizure |
| G40.2 | Localization-related (focal) (partial) symptomatic epilepsy and epileptic syndromes with complex partial seizures [G40.2] | Focal seizure |
| G40.20 | Localization-related (focal) (partial) symptomatic epilepsy and epileptic syndromes with complex partial seizures, not intractable [G40.20] | Focal seizure |
| G40.201 | Localization-related (focal) (partial) symptomatic epilepsy and epileptic syndromes with complex partial seizures, not intractable, with status epilepticus [G40.201] | Focal seizure_SE |
| G40.209 | Localization-related (focal) (partial) symptomatic epilepsy and epileptic syndromes with complex partial seizures, not intractable, without status epilepticus [G40.209] | Focal seizure |
| G40.21 | Localization-related (focal) (partial) symptomatic epilepsy and epileptic syndromes with complex partial seizures, intractable [G40.21] | Focal seizure |
| G40.211 | Localization-related (focal) (partial) symptomatic epilepsy and epileptic syndromes with complex partial seizures, intractable, with status epilepticus [G40.211] | Focal seizure_SE |
| G40.219 | Localization-related (focal) (partial) symptomatic epilepsy and epileptic syndromes with complex partial seizures, intractable, without status epilepticus [G40.219] | Focal seizure |
| G40.3 | Generalized idiopathic epilepsy and epileptic syndromes [G40.3] | Generalized |
| G40.30 | Generalized idiopathic epilepsy and epileptic syndromes, not intractable [G40.30] | Generalized |
| G40.301 | Generalized idiopathic epilepsy and epileptic syndromes, not intractable, with status epilepticus [G40.301] | Generalized_SE |
| G40.309 | Generalized idiopathic epilepsy and epileptic syndromes, not intractable, without status epilepticus [G40.309] | Generalized |
| G40.31 | Generalized idiopathic epilepsy and epileptic syndromes, intractable [G40.31] | Generalized |
| G40.311 | Generalized idiopathic epilepsy and epileptic syndromes, intractable, with status epilepticus [G40.311] | Generalized_SE |
| G40.319 | Generalized idiopathic epilepsy and epileptic syndromes, intractable, without status epilepticus [G40.319] | Generalized |
| G40.4 | Other generalized epilepsy and epileptic syndromes [G40.4] | Generalized |
| G40.40 | Other generalized epilepsy and epileptic syndromes, not intractable [G40.40] | Generalized |
| G40.401 | Other generalized epilepsy and epileptic syndromes, not intractable, with status epilepticus [G40.401] | Generalized_SE |
| G40.409 | Other generalized epilepsy and epileptic syndromes, not intractable, without status epilepticus [G40.409] | Generalized |
| G40.41 | Other generalized epilepsy and epileptic syndromes, intractable [G40.41] | Generalized |
| G40.411 | Other generalized epilepsy and epileptic syndromes, intractable, with status epilepticus [G40.411] | Generalized_SE |
| G40.419 | Other generalized epilepsy and epileptic syndromes, intractable, without status epilepticus [G40.419] | Generalized |
| G40.5 | Epileptic seizures related to external causes [G40.5] | Other |
| G40.50 | Epileptic seizures related to external causes, not intractable [G40.50] | Other |
| G40.501 | Epileptic seizures related to external causes, not intractable, with status epilepticus [G40.501] | Other_SE |
| G40.509 | Epileptic seizures related to external causes, not intractable, without status epilepticus [G40.509] | Other |
| G40.8 | Other epilepsy and recurrent seizures [G40.8] | Other |
| G40.80 | Other epilepsy [G40.80] | Other |
| G40.801 | Other epilepsy, not intractable, with status epilepticus [G40.801] | Other_SE |
| G40.802 | Other epilepsy, not intractable, without status epilepticus [G40.802] | Other |
| G40.803 | Other epilepsy, intractable, with status epilepticus [G40.803] | Other_SE |
| G40.804 | Other epilepsy, intractable, without status epilepticus [G40.804] | Other |
| G40.81 | Lennox-Gastaut syndrome [G40.81] | Other |
| G40.811 | Lennox-Gastaut syndrome, not intractable, with status epilepticus [G40.811] | Other_SE |
| G40.812 | Lennox-Gastaut syndrome, not intractable, without status epilepticus [G40.812] | Other |
| G40.813 | Lennox-Gastaut syndrome, intractable, with status epilepticus [G40.813] | Other_SE |
| G40.814 | Lennox-Gastaut syndrome, intractable, without status epilepticus [G40.814] | Other |
| G40.82 | Epileptic spasms [G40.82] | Other |
| G40.821 | Epileptic spasms, not intractable, with status epilepticus [G40.821] | Other_SE |
| G40.822 | Epileptic spasms, not intractable, without status epilepticus [G40.822] | Other |
| G40.823 | Epileptic spasms, intractable, with status epilepticus [G40.823] | Other_SE |
| G40.824 | Epileptic spasms, intractable, without status epilepticus [G40.824] | Other |
| G40.89 | Other seizures [G40.89] | Other |
| G40.9 | Epilepsy, unspecified [G40.9] | Unspecified |
| G40.90 | Epilepsy, unspecified, not intractable [G40.90] | Unspecified |
| G40.901 | Epilepsy, unspecified, not intractable, with status epilepticus [G40.901] | Unspecified_SE |
| G40.909 | Epilepsy, unspecified, not intractable, without status epilepticus [G40.909] | Unspecified |
| G40.91 | Epilepsy, unspecified, intractable [G40.91] | Unspecified |
| G40.911 | Epilepsy, unspecified, intractable, with status epilepticus [G40.911] | Unspecified_SE |
| G40.919 | Epilepsy, unspecified, intractable, without status epilepticus [G40.919] | Unspecified |
| G40.A | Absence epileptic syndrome [G40.A] | Other |
| G40.A0 | Absence epileptic syndrome, not intractable [G40.A0] | Other |
| G40.A01 | Absence epileptic syndrome, not intractable, with status epilepticus [G40.A01] | Other_SE |
| G40.A09 | Absence epileptic syndrome, not intractable, without status epilepticus [G40.A09] | Other |
| G40.A1 | Absence epileptic syndrome, intractable [G40.A1] | Other |
| G40.A11 | Absence epileptic syndrome, intractable, with status epilepticus [G40.A11] | Other_SE |
| G40.A19 | Absence epileptic syndrome, intractable, without status epilepticus [G40.A19] | Other |
| G40.B | Juvenile myoclonic epilepsy [impulsive petit mal] [G40.B] | Other |
| G40.B0 | Juvenile myoclonic epilepsy, not intractable [G40.B0] | Other |
| G40.B01 | Juvenile myoclonic epilepsy, not intractable, with status epilepticus [G40.B01] | Other_SE |
| G40.B09 | Juvenile myoclonic epilepsy, not intractable, without status epilepticus [G40.B09] | Other |
| G40.B1 | Juvenile myoclonic epilepsy, intractable [G40.B1] | Other |
| G40.B11 | Juvenile myoclonic epilepsy, intractable, with status epilepticus [G40.B11] | Other_SE |
| G40.B19 | Juvenile myoclonic epilepsy, intractable, without status epilepticus [G40.B19] | Other |
| R56 | Convulsions, not elsewhere classified | Seizure_Convulsion |
| R5600 | Simple febrile convulsions | Seizure_Convulsion |
| R561 | Post traumatic seizures | Seizure_Convulsion |
| R560 | Febrile convulsions | Seizure_Convulsion |
| R5601 | Complex febrile convulsions | Seizure_Convulsion |
| R569 | Unspecified convulsions | Seizure_Convulsion |
| G25.3 | Myoclonus | Seizure_Convulsion |
