# Supplementary material for: Epidemiology and injectable antiseizure medication treatment patterns of seizure patients treated in United States hospitals
Source: Front Neurol. 2022 Sep 12;13:941775. doi: 10.3389/fneur.2022.941775 (PMC9510892; doi:10.3389/fneur.2022.941775)
Supplement: Supplementary file 3 [file Table_2.DOCX]

**eTable 2. ICD-10 Codes for St. Germaine-Smith Comorbidities**

| Congestive Heart Failure | ICD-9: 398.91, 402.01, 402.11, 402.91, 404.01, 404.03, 404.11, 404.13, 404.91, 404.93, 425.4, 425.5, 425.7, 425.8, 425.9, 428%  ICD-10: I09.81, I09.9, I11.0, I13.0, I13.2, I25.5, I42.0, I42.5, I42.6, I42.7, I42.8, I42.9, I43%, I50%, P29.0 |
| --- | --- |
| Peripheral Vascular Disease | ICD-9: 093.0, 437.3, 440%, 441%, 443.1, 443.2%, 443.8%, 443.9, 447.1, 557.1, 557.9, V43.4  ICD-10: I70%, I71%, I73.1, I73.8, I73.9, I77.1, I79.0, I79.2, K55.1, K55.8, K55.9, Z95.8, Z95.9 |
| Moderate or Severe Renal Disease | ICD-9: 403.01, 403.11, 403.91, 404.02, 404.03, 404.12, 404.13, 404.92, 404.93, 582%, 583%, 585%, 586%, 588.0, V42.0, V45.1, V56  ICD-10: I12.0, I13.1, N03.2, N03.3, N03.4, N03.5, N03.6, N03.7, N05.2, N05.3, N05.4, N05.5, N05.6, N05.7, N18%, N19%, N25.0, Z49.0, Z49.1, Z49.2, Z94.0, Z99.2 |
| Moderate or Severe Liver Disease | ICD-9: 456.0, 456.1, 456.2, 572.2, 572.3, 572.4, 572.8ICD-10: I85.0, I85.9, I86.4, I98.2, K70.4, K71.1, K72.1, K72.9, K76.5, K76.6, K76.7 |
| Metastatic cancer | ICD-9 : 196%, 197%, 198%, 199%  ICD-10 : C77%, C78%, C79%, C80% |
| Brain Tumor | ICD-9 : 191%, 192%, 194.3, 194.4, 200.5, 225.0, 225.1, 225.2, 225.8, 225.9, 227.3, 227.4, 237.0, 237.1, 237.5, 237.6, 237.7, 237.9, 239.6  ICD-10 : C70%, C71%, C72%, C75.1, C75.2 C75.3, D33.0, D33.1, D33.2, D33.7, D33.9, D35.2, D35.3, D35.4, D42.0, D42.9, D43.0, D43.1, D43.2, D43.3, D43.7, D43.9, D44.3, D44.4, D44.5 |
| Solid Tumor without Metastasis | ICD-9: 140, 141, 142, 143, 144, 145, 146, 147, 148, 149, 150, 151, 152, 153, 154, 155, 156, 157 ,158, 159, 160, 161, 162, 163, 164, 165, 166, 167, 168, 169, 170, 171, 172, 174, 175, 176, 177, 178, 179, 180, 181, 182, 183, 184, 185, 186, 187, 188, 189, 190, 193, 194.0, 194.1, 194.5, 194.6, 194.8, 194.9, 195  ICD-10 : C00%, C01%, C02%, C03%, C04%, C05%, C06%, C07%, C08%, C09%, C10%, C11%, C12%, C13%, C14%, C15%, C16%, C17%, C18%, C19%, C20%, C21%, C22%. C23%. C24%. C25%, C26%, C30%, C31%, C32%, C33%, C34%, C37%, C38%, C39%, C40%, C41%, C43%, C45%, C46%, C47%, C48%, C49%, C50%, C51%, C52%, C53%, C54%, C55%, C56%, C57%, C58%, C60%, C61%, C62%, C63%, C64%, C65%, C66%, C67%, C68%, C69%, C73%, C74%, C75% C76%, C97 |
| Paraplegia and Hemiplegia | ICD-9: 334.1, 342.0, 342.1, 342.8, 342.9, 344.0, 344.1, 344.2, 344.3, 344.4, 344.5, 344.6, 344.9  ICD-10: G81.%, G82.%, G04.1, G11.4, G83.0, G83.1, G83.2, G83.3, G83.4, G83.9 |
| Aspiration Pneumonia | ICD-9: 507.0  ICD-10 : J69.0 |
| Dementia | ICD-9: 290%, 294.1, 331.2  ICD-10: F00%, F01%, F02%, F03%, F05, G30%, G31.1 |
| Pulmonary Circulation Disorders | ICD-9: 415.0, 415.1, 416%, 417.0, 417.8, 417.9  ICD-10: I26%, I27%, I28.0, I28.8, I28.9 |
| Cardiac Arrhythmias | ICD-9: 426.0, 426.13, 426.7, 426.9, 426.10, 426.12, 427.0, 427.1, 427.2, 427.3, 427.4, 427.6, 427.8, 427.9, 785.0, 996.01, 996.04, V45.0, V53.3  ICD-10: I44.1 I44.2, I44.3, I45.6, I45.9, I47%, I48%, I49%, R00.0, R00.1, R00.8, T82.1, Z45.0, Z95.0 |
| Hypertension | ICD-9: 401%, 402%, 403%, 404%, 405%  ICD-10: I10%, I11%, I12%, I13%, I15% |
| Anoxic Brain Injury | ICD-9: 348.1, 768.5, 768.6, 768.7, 768.9  ICD-10: G93.1, P21%, P91.6% |
