# Supplementary material for: Epidemiology and injectable antiseizure medication treatment patterns of seizure patients treated in United States hospitals
Source: Front Neurol. 2022 Sep 12;13:941775. doi: 10.3389/fneur.2022.941775 (PMC9510892; doi:10.3389/fneur.2022.941775)
Supplement: Supplementary file 4 [file Table_3.DOCX]

eTable 3. Injectable Anti-Seizure Medications

| **Generic Drug Name** | **Brand Drug Name** | **Route/Dosage Form** |
| --- | --- | --- |
| Brivaracetam | Briviact | Oral – tablet, suspension; intravenous |
| Fosphenytoin | Cerebyx | Intravenous |
| Lacosamide | Vimpat | Oral – tablet; intravenous |
| Levetiracetam | Keppra | Oral – tablet, solution; intravenous |
| Phenobarbital | Solfoton, Luminal | Oral – tablet, intramuscular, intravenous |
| Valproate sodium | Depacon | Intravenous |
| **Benzodiazepine** |  |  |
| Midazolam | Versed | Intravenous |
| Diazepam | Valium | Intravenous |
| Lorazepam | Ativan | Intravenous |
